# Humanizing care in the ICU: how system pressures shape interacting patterns of hurry, blur, burden, and cruelty

**DOI:** 10.3389/frhs.2026.1867800

**Published:** 2026-08-11

**Authors:** Misk Al Zahidy, Ana Gabriela Claros, Katerina Mulanovich, Mohamed A. Mahmoud, Michael R. Gionfriddo, Maria Jose Lizarazo, Maria Mateo Chavez, Ahmed Sayed Ahmed, Yue Dong, Ognjen Gajic, Victor M. Montori, Sumera Ahmad

**Affiliations:** 1 Knowledge and Evaluation Research Unit, Division of Endocrinology, Diabetes, Metabolism and Nutrition, Mayo Clinic, Rochester, MN, United States; 2 Division of Pulmonary, Critical Care, Allergy and Sleep Medicine, Mayo Clinic, Rochester, MN, United States

**Keywords:** artificial intelligence, clinical workload, communication, intensive care unit, pathologies of care, patient-centered care, qualitative research, treatment burden

## Abstract

**Background:**

Intensive care units are high-acuity environments characterized by time pressure, medical complexity, and organizational demands. These conditions may influence clinicians’ ability to provide patient-centered care and contribute to forms of care perceived as fragmented or dehumanizing.

**Objective:**

To examine how ICU clinicians describe and make sense of the pressures shaping everyday critical care practice, and how these pressures give rise to interacting patterns of care, including those described as hurry, blur, burden, and cruelty.

**Methods:**

We conducted a qualitative study using focus groups with ICU clinicians from a large academic health system. Discussions were informed by the concept of the pathologies of care as sensitizing concepts. Four focus groups were held with physicians, nurses, advanced practice nurses, and a social worker (*N* = 14). Transcripts were analyzed using directed content analysis with both deductive and inductive coding.

**Results:**

Clinicians described these patterns not as discrete phenomena but as interrelated, context-dependent processes shaped by shared operational pressures such as time constraints, workload, and information demands. These dynamics were often viewed as inherent to the structure and culture of ICU care and were described as co-occurring and compounding. Communication emerged as both a contributor to and a potential mechanism for mitigation, alongside system-level strategies such as reducing documentation burden and leveraging artificial intelligence to support care delivery.

**Conclusions:**

Patterns reflected in hurry, blur, burden, and cruelty are best understood as emergent properties of complex care systems. Addressing these dynamics may require system-level approaches to improve communication, reduce workload pressures, and support more patient-centered, humanizing care.

## Introduction

Patient-centered care responds to the problematic situation of the patient, advancing it in ways that patients themselves find worthwhile and as minimally disruptive as possible of their lives and relationships (1). This approach includes practices that enable clinicians to notice people beyond their physiological abnormalities, establish meaningful communication and relationships, and support policies that facilitate individualized care (2). The concept of “care that fits” further emphasizes responsiveness to patients’ values, goals, and life context, highlighting the importance of minimizing treatment burden while preserving personhood.

Pressures of demand, access, costs, and efficiency have led healthcare systems to become increasingly industrialized (3). When care becomes industrialized, it can shift toward the processing of patients and their data failing to respond to each patient's problematic situation in ways that mobilize both competence and compassion, or that patients would find adequate and desirable. Such responses are neither careful—because they do not offer evidence-based care tailored to the individual—nor kind, as they may disregard the time, energy, and attention of patients and their families (4, 5).

These challenges may be particularly evident in intensive care settings, where the demands of high acuity illness, technological complexity, and urgent clinical decision making are most pronounced (6, 7). The intensive care unit (ICU) is a time-sensitive environment in which critically ill patients require life sustaining interventions and continuous monitoring. Patients often experience rapidly changing clinical conditions, requiring clinicians to make prompt decisions while interpreting large volumes of information and managing complex technological systems (6, 7).

In addition to these technical demands, studies examining intensive care experiences report significant relational and emotional challenges (8, 9). Patients and families frequently describe ICU care as isolating, disorienting, and at times dehumanizing, particularly when communication is insufficient or when care becomes primarily task oriented (8, 9). Failures in respectful care, including communication breakdowns and lack of attention to patient dignity, have been associated with emotional distress and negative psychological outcomes for patients and relatives (10). Research on compassion in intensive care further highlights tensions between technological demands and the provision of patient-centered, humanizing care (11).

Despite growing research examining humanizing and dehumanizing experiences in intensive care, most studies have focused on patients’ and families’ perspectives, with comparatively less attention to clinicians’ own experiences in navigating these competing demands (8, 9).

While these pressures have been examined across multiple domains in ICU research, they are often studied in isolation (e.g., workload, burnout, communication failures, or depersonalization) (8–16). Less attention has been given to how clinicians themselves make sense of these pressures as part of everyday practice, and how they may co-occur and interact to shape care delivery. Understanding these dynamics is essential for informing the design of health systems that support patient-centered, high-value care.

The concept of “pathologies of care” (hurry, blur, burden, and cruelty) has been proposed to describe patterns that emerge in industrialized healthcare systems, but has largely remained theoretical and unexamined in ICU settings (3–5). Rather than treating these pathologies as fixed categories or a formal evaluative framework, they can be understood as sensitizing concepts that help illuminate how clinicians interpret and describe their experiences in practice.

Therefore, our objective was to examine how ICU clinicians describe and make sense of the pressures that give rise to interacting patterns of care, including those described as hurry, blur, burden, and cruelty.

## Materials and methods

We conducted a qualitative study using focus groups to explore ICU clinicians’ experiences and perspectives regarding how pressures shaping everyday critical care practice influence clinicians’ ability to provide patient-centered care in the ICU. Study reporting followed the Standards for Reporting Qualitative Research (SRQR) (17).

The discussion was informed by the concept of the pathologies of care (hurry, blur, burden, and cruelty) as a sensitizing lens, while allowing clinicians to describe their experiences freely (3). Focus groups were selected because they allow participants to build on one another's experiences and collectively reflect on shared practices, which is particularly useful for examining complex, socially embedded aspects of ICU work. This approach allowed us to capture how clinicians co-construct interpretations of care practices in real-world settings, which may not emerge through surveys or individual interviews.

Eligible participants were adult ICU clinicians actively working at Mayo Clinic in Rochester, Minnesota or affiliated Mayo Clinic Health System sites. They were identified using the institutional directory. Potential participants received an email invitation that described the purpose of the study and included the date and time of the focus group session if they were interested in participating. To promote diverse perspectives, we also used a snowball sampling approach in which invited clinicians were encouraged to share the study information with eligible colleagues.

We aimed to achieve variability in participant characteristics to capture a more comprehensive understanding of different experiences and perspectives by inviting all ICU healthcare professionals. All sessions were conducted virtually via Microsoft Teams between November 2024 and February 2025 and were facilitated by a trained qualitative researcher (MRG) with a semi-structured discussion guide (Supplementary Material S1).

The focus group guide included open-ended questions about exploring how clinicians experience the pressures that shape everyday ICU practice, drawing on the concept of the pathologies of care while allowing for emergent perspectives, and potential strategies to mitigate them. Discussions were audio-recorded and transcribed using Microsoft Teams. Transcripts were reviewed and revised by the study team for accuracy. After each session, clinicians completed a brief demographic questionnaire via REDCap to collect professional background information (e.g., role, years of ICU experience).

Recruitment continued until data saturation was reached, defined as the point at which no new themes or variations in how clinicians described the pressures shaping ICU care or their interrelationships were identified.

### Data analysis

Transcripts were analyzed using directed content analysis, informed by an initial coding framework based on the four pathologies of care. These constructs were used as sensitizing concepts rather than fixed analytic categories, allowing for both deductive and inductive coding. We selected this qualitative approach because the pathologies of care have been proposed conceptually but have not been examined empirically within ICU settings, allowing us to explore how clinicians interpret, apply, question, or expand upon these constructs in practice.

A preliminary codebook was developed using this framework, with additional inductive codes added based on data collected during the focus groups. Three trained team members (MRG, KM, AGC) independently coded each transcript. Discrepancies were resolved through discussion until consensus was reached. Themes were iteratively refined through team meetings to ensure rigor and consistency in interpretation. The focus groups were facilitated by MRG, who has training and experience in qualitative research, and transcripts were coded by MRG, KM, and AGC, who had training or experience in qualitative analysis. The research team included clinicians and health services researchers with backgrounds in critical care and patient-centered care, and several members were familiar with the pathologies-of-care concepts that informed the study.

### Ethical considerations

The Mayo Clinic Institutional Review Board approved all study procedures (IRB #23-010100). Verbal consent was obtained from all participants. All data were de-identified during analysis to maintain participant confidentiality.

## Results

Four focus group sessions were held between November 2024 and February 2025, with 3–4 participants per group (Table 1). Participants included ICU physicians, nurses, and nurse practitioners with a median of 11.5 years of ICU experience. Each session lasted approximately 60 min.

**Table 1:** Baseline characteristics of focus group participants.

Characteristic	Participants *N* = 14
Gender, *n*	
Female	9
Male	5
Clinician role, *n*	
Clinical nurse specialist	2
Nurse practitioner	1
Physician	10
Social worker	1
Years of experience in clinical practice, median (IQR)	11.5 (15)

IQR, Interquartile range.

Our analysis identified how clinicians describe the pressures shaping everyday ICU practice and how these pressures gave rise to patterns of care in real-world settings. Rather than describing these patterns as discrete or independent phenomena, clinicians consistently characterized them as interrelated processes emerging from shared operational pressures (e.g., time constraints, workload, and information demands). These patterns, at times resembling hurry, blur, burden, and cruelty, were described as dynamic, context-dependent, and occasionally necessary responses to clinical situations.

Clinicians described numerous instances and noted that some responses, though seemingly pathologic, may be appropriate in certain situations. Clinicians emphasized how the ICU's nature, policies, and culture foster these patterns and how they often interconnect and compound. Communication emerged as both a contributor to and a potential remedy. Finally, clinicians discussed mitigation strategies, including reducing documentation and using artificial intelligence (AI). Quotes are presented in text; additional examples appear in Table 2.

**Table 2:** Pathologies of care and illustrative examples.

Pathology	Description	Exemplary quotes
Hurry	Occurs when the pace care adopts is too fast relative to the pace that would have been necessary to meet the demands of the situation.	“…the other aspect of hurrying is that I have noticed in my practice that we try our best to teach residents, to have teaching sessions every afternoon, unless there's some calamity, we try to have some kind of somebody give a talk. So, didactics is always prioritized, but I've never seen anybody emphasize ok, you have a free hour right now, there's no admissions, no calamity, no cheating, why don't you go and spend some time at bedside? Like, talk to your patient? Socialize with them, get to know them a little bit. That's not a practice at all that I've been taught, or that I have seen, and it takes a lot of effort to try to get the residents to actually go back into the patient room when they don't have to poke them with the stethoscope.” (Participant 1, Physician)
Blur	Occurs when clinicians cannot notice patients and appreciate their situation in sufficient detail because they see patients as their abnormal test results, as members of a statistical group (e.g., poor prognosis), or exclusively through the content of the medical record.	…during our medical training, that was never emphasized as important. Our focus is all on physiology and disease management. We are never connected to human beings as a whole… it's really hard to see people any other way than whatever illness they are there with.it takes either personal experience, or some other motivation to look at things differently. (Participant 1, Physician) So even though we talk about emotional isolation. I think that it's much more healthy to have those emotions and even discuss those emotions with the family members or with the patient so that there's a lot of empathy, not just sympathy, … when I was training in medical school, they told me I shouldn't be doing. And I have found that that was wrong, that we should be as empathetic as possible. (Participant 11, Physician)
Burden	The distress that results when the workload of accessing and enacting care exceeds the available capacity of the individual to shoulder it.	“I've heard from patients that they just feel overwhelmed by the number of health care providers, by the information, and by the decision making” (Participant 5, Physician)
Cruelty	Occurs when patients seeking care receive indifference, when their needs and humanity are incidentally overlooked due to institutional structures or policies, when they receive labels that exclude them from care (e.g., noncompliant), or when they are perceived as means to an end.	If you… create a situation where a person's less than human, less than your equal, then it facilitates all sorts of wrongs…or not human….So, are they human? Are they machines? If they're… completely supported by all of our technology… is he man? Is he a machine? …it makes it easier to be cruel… when you start to dehumanize people. (Participant 3, APRN)

### Perception of pathologies

Clinicians described numerous instances of the pathologies of care in the ICU, but also recognized that some responses, though seemingly pathologic, may be appropriate. For example, acting hurriedly during an acute event may be necessary, even if the patient remains a blur. In such cases, rapid action is not pathological. However, outside these contexts, haste may lead to indifference toward patient and family needs.

Clinicians emphasized that the appropriateness of these responses depended on the phase of care, with patterns such as hurry being valued during acute stabilization but perceived negatively when sustained beyond that context.

I also would add it depends on what part of the care that we're in. If we're at the beginning… the hurrying is appreciated…The family wants you to hurry. The patient wants you to hurry. They want you to do these things quickly to get the outcome that everybody's looking for. I would say later in their care, in their ICU care, hurry is seen as more of a negative because… if somebody feels like they're coming in to be talked to and that provider is in a hurry, then that's seen as negatively (Participant 13, APRN)

### ICU nature and culture

The ICU's unpredictability makes advance scheduling difficult. Emergencies or new patient needs may overwhelm available time, prompting clinicians to act hastily to keep pace. This may result in inappropriate responses to patient needs.

I sort of feel like there is a time pressure from the first second I walk in, in the morning because you're on the clock in terms of doing the routine work, rounding on the patients, looking at the labs, getting the baseline work of the day done before rounds. (Participant 8, Physician)

The ICU's unpredictability and the high illness burden of patients often create practical, cognitive, and emotional burdens for clinicians, frequently described as feeling overwhelmed. Clinicians also noted that patients may be overwhelmed by the number of clinicians, the volume of information and decisions, and the sensory load from alarms and visual displays.

These pressures were described as arising not only from individual workload, but from the structure and organization of ICU care, including frequent admissions and discharges, coordination demands, and the need for continuous vigilance.

Clinicians described how these system-level demands compress time, requiring rapid decision-making while simultaneously managing complex care, team coordination, and emotionally difficult situations (e.g., death), contributing to a persistent sense of overload.

To reiterate from what people have said about the burden… I don't know if burden is the right word *per se*, but like you get to know the family, you get to know the patient and you see their ups and downs and all of a sudden something happens that also takes a toll and part of the job when it's necessary. (Participant 7, Physician)

Beyond burden, the ICU's nature may also contribute to blur, both physical and emotional. Patients with critical illness often require aggressive interventions, which can lead to physical blurring of the patient.

…what blurs the patient … I think about how technical the care has become, how many devices you can hardly get to some of our patients, literally like in the bed, you can hardly like get to them physically because of the number of devices that are connected to them. And it’s quite literally a blurring of the boundary between the technical and the human (Participant 3, APRN)

Clinicians also described blur as both a consequence of the technological environment and, at times, an adaptive response to the emotional and cognitive demands of ICU care. However, some emphasized the importance of maintaining empathy (Table 2).

In addition to burden and blur, clinicians noted that the nature of the ICU can lead to cruelty, especially when responses feel indifferent to the patient’s situation or to the family’s understanding of what is happening.

… critical care is invasive and uncomfortable. We do our best to try to mitigate those things, but the tubes and lines …are by definition pretty aggressive. I think… when you talk about cruelty in the ICU is situations where we find ourselves delivering these aggressive and invasive interventions to people who have little to no chance of survival. (Participant 8, Physician)

#### Culture of practice

Clinicians noted that medical training culture may contribute to blur by prioritizing didactics away from the bedside. An emphasis on physiologic and task-oriented aspects of care may shift attention away from the patient as a person, leaving emotional, psychological, and social needs less visible (Table 1).

### Pathologies as connected

**Figure 1:**
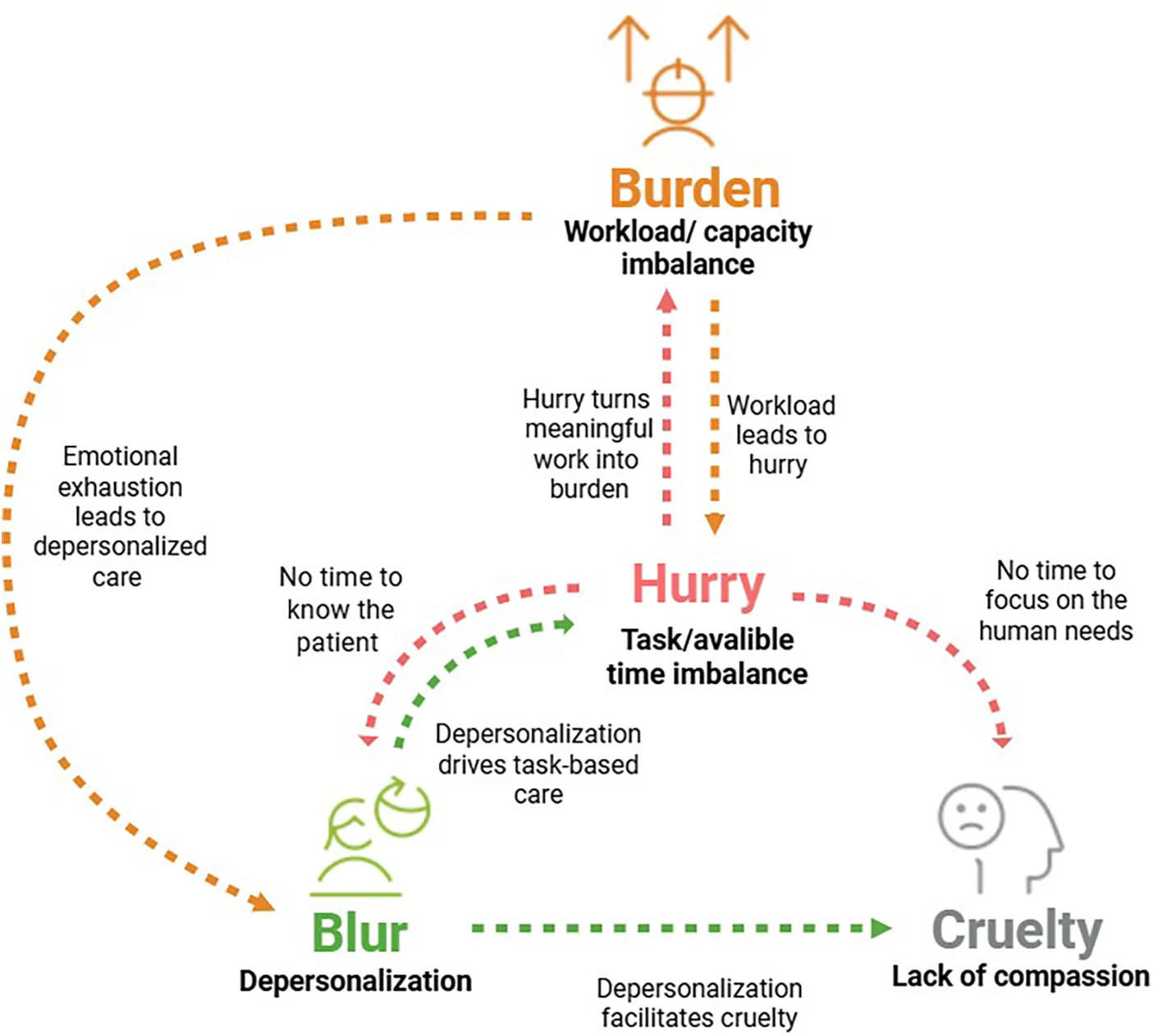
Interconnection of care pathologies in the ICU. Color-coded icons identify Burden (orange), Hurry (pink), Blur (green), and Cruelty (grey). In this figure, each term refers to its pathologic expression: burden as workload exceeding available capacity; hurry as a pace disproportionate to the needs of the situation or persisting beyond necessary clinical urgency; blur as failure to perceive the patient and their situation in sufficient detail; and cruelty as indifference to patients’ needs or humanity. Dashed arrows depict relationships described by clinicians among these pathologic patterns.

Clinicians consistently described these patterns as interconnected processes rather than isolated experiences, with some patterns directly contributing to others (Figure 1).

Burden and hurry were closely linked; clinicians felt hurried when the workload exceeded available time. They also described how hurry could turn meaningful work into something burdensome.

It's not just the paperwork and documentation…but also having you know to teach and have the patient care, family meeting, teaching at different levels the residents, the fellows. Having to combine all of that with the time constraint, all can be perceived as a burden, although you actually do enjoy doing all of it… it's just with the time constraint something pleasurable becomes a burden. (Participant 5, Physician)

Clinicians also described how hurry limited their ability to understand what matters to the patient (i.e., blur), which could lead to cruelty through dehumanization. Hurry could also cause omissions in care (e.g., missed labs needing re-draws).

These descriptions illustrate how multiple pressures interact to shape care delivery, reinforcing one another rather than occurring independently (Table 2).

### Communication as contributor to pathologies

Clinicians across all focus groups emphasized the importance of communication in ICU care. Yet they acknowledged that communication is often challenging, and its breakdown contributes to the pathologies of care. In critical illness, everyone seeks rapid answers, which can abbreviate communication. In-depth exchanges may be deprioritized in favor of medical needs, causing fear, anxiety, blur (e.g., not understanding what matters), and even cruelty. As one participant described:

I handed off the service and the new consultant was brand new. … it was a time of shift change when a patient was very sick., the patient passed away and nobody had yet talked to the family by the time the coroner approached them about conducting an autopsy, and they didn't even know the patient had passed away… it was very cruel that this is the first thing you're asking the family. (Participant 10, Physician)

Clinicians frequently mentioned documentation as a key contributor to pathologies. It directly increased hurry and burden by adding tasks in a time-constrained setting and indirectly led to blur and cruelty by reducing time with patients.

### Mitigating ICU pathologies of care

Since poor communication contributes to all pathologies, clinicians emphasized that improving it could mitigate them and enhance patient and family experiences in the ICU. For instance, introducing themselves or setting expectations helped reduce miscommunication, especially in hurried situations.

I and other team members I've seen have made a conscious note to the patient that, hey, things are going to be hectic for a little bit. We're going… to get things stabilized and then we will have time to sit down and talk …. I think the perception of hurrying that is provided to the patient is something that's voluntary, but the hurry exists separately, so sometimes people are able to kind of not show that hurry. (Participant 12, Physician)

Additionally, to mitigate the hurry and blur, clinicians highlighted the value of pausing at key moments in patient care to prevent these pathologies.

…if you take 15 s to say, does this person, who are they as a person before you start chest compressions.It's even more important to know who they are as a person to know if they, if this is what they want, especially when you don't know them, I don't think you need their favorite color and their favorite book and their favorite kind of music. But a quick question of what do they do? …What brings value to them as a person? (Participant 9, APRN)

Further, clinicians described the “Get to Know Me Board,” a tool that helps them learn about the patient as a person. It includes personal details like nicknames, photos, hobbies, and relationships (18, 19). This board illustrates how institutional practices can help prevent care pathologies.

Reducing required documentation was also identified as a strategy to mitigate hurry and burden.

But if we can fix the fixable things like the documentation aspect of it, or the time in part of the computer, that may help to leave some of the other stuff that may not be the only solution, but maybe part of the solution. (Participant 7, Physician)

#### Use of AI

To reduce hurry and burden in the ICU, clinicians recommended AI to reduce workload and enhance their capacity to care, for example, through tools that assist with documentation and clinical decision support.

I mean, we're starting to investigate tools like ambient listening, large language models, NLPs to help us with those tools of sorting through the charts, and all the information that patients have. And the more we can leverage and rely on tools …The more time we can focus on patients and ensuring we're going down the right path for that patient. (Participant 9, APRN)

#### Thoughtful self-reflection

Finally, clinicians discussed how being thoughtful and mindful of each patient’s needs, through self-reflection, can help mitigate the pathologies of care.

…reminding ourselves, why are we doing what we're doing in the first place? Sometimes it may help to reflect and step back and see what we have become. And maybe that's not why we went there in that profession. (Participant 1, Physician)

## Discussion

In this qualitative study, ICU clinicians described how pressures inherent to everyday critical care practice may give rise to patterns reflected in the pathologies of care: hurry, burden, blur, and cruelty. Participants reported experiencing these patterns in daily ICU work and emphasized that they often arise from the nature and culture of the ICU, including unpredictability, high illness burden, technological intensity, and time pressure. Clinicians also noted that some responses that may appear to be pathologic, such as acting quickly during acute deterioration, can be appropriate in specific contexts. Importantly, these pathologies were described as dynamic and context-dependent, where workload and time pressure contribute to hurry and burden, which may in turn lead to blur and occasionally cruelty. Communication and other practices, such as reducing documentation burden and reflective practice, were identified as potential strategies to mitigate these patterns. This study is among the first to empirically examine pathologies of care in ICU settings from the clinician perspective.

While prior ICU research has documented workload, depersonalization, and communication challenges, our findings suggest that clinicians experience these not as isolated issues but as dynamically interacting processes. By examining these pressures through clinicians’ accounts, this study offers a process-oriented perspective on how systemic demands translate into patterns of care that may be experienced as hurried, fragmented, or dehumanizing. This shifts the interpretation of these challenges from isolated deficits (e.g., burnout or poor communication) toward a systems-level understanding of how multiple pressures interact to shape care delivery.

Rather than functioning as a fixed conceptual framework, the pathologies of care in this study operated as sensitizing concepts that helped illuminate how clinicians describe and interpret their experiences. Our findings suggest that these patterns are best understood as emergent properties of complex care systems, rather than discrete categories to be identified.

Several pressures described by clinicians in this study have been previously reported in ICU literature. Workload, competing clinical demands, and time pressure have been associated with challenges in maintaining patient-centered interactions in intensive care settings (12, 13, 20). Clinicians in our study described how these pressures are experienced in practice as hurry and burden during everyday ICU work. Prior studies have also documented depersonalization and emotional distancing among ICU clinicians exposed to repeated suffering and high-acuity illness (14, 15). Participants reflected on how similar dynamics may manifest as blur when technological demands and clinical priorities shift attention away from the patient as a person. Research examining humanizing and dehumanizing ICU care has further highlighted how communication failures and organizational pressures shape patients’ and families’ experiences (8, 9). Our findings add the clinicians’ perspective by illustrating how these same conditions may contribute to responses perceived as cruel when hurried or fragmented communication limits attention to patients and families. Clinicians also described how these dynamics often interact and reinforce one another. Burden and hurry frequently contributed to blur, a process resembling the depersonalization previously described among ICU clinicians (14, 15), and together these dynamics could lead to responses perceived as cruel (16).

Participants identified several strategies that may help mitigate these patterns. Efforts to humanize care, such as “Get to Know Me” boards (18, 19, 21), patient photographs (22), and other patient-centered practices (23), may help clinicians recognize patients as persons and counteract blur, allowing clinicians to see patients in “high definition” (24). Communication also emerged as a central mechanism through which these dynamics may be mitigated, consistent with prior work highlighting its role in humanized ICU care (25–27). Participants further suggested that reducing documentation burden and using tools such as artificial intelligence to support information synthesis may allow clinicians to dedicate more time to direct patient care (28).

This study has several strengths. Four focus groups were conducted, allowing clinicians to share and compare their experiences and reflect on one another's perspectives (29). The small size of each group facilitated in-depth discussion and provided participants with additional time to describe their experiences in detail. The focus group format also enabled clinicians to build on one another's reflections, enriching the discussion of complex aspects of ICU care (29). Importantly, participants openly discussed sensitive topics, including situations perceived as cruel in ICU practice. In addition, the study included clinicians from different professional roles, allowing perspectives from multiple positions within ICU care to be represented. Data saturation was reached after four focus groups, a sample size often considered sufficient in qualitative research (30).

Despite these contributions, several limitations should be considered. Participants were recruited from a single academic health system, which may limit the transferability of findings to other ICU settings with different organizational structures. Participation was voluntary and depended on clinicians’ availability to attend scheduled focus group sessions, which may have limited participation from nurses, nurse practitioners, and social workers who often work shift-based schedules. Because different professional groups may experience organizational pressures and patient care responsibilities differently, this imbalance may have limited the range of perspectives captured in the discussions. In addition, conducting focus groups with mixed professional roles may have influenced participation dynamics, particularly given hierarchical structures in ICU settings. Although facilitators encouraged equitable participation, some perspectives, especially from non-physician clinicians, may have been underrepresented.

Future research should further examine the role of culture, experience, teamwork, and organizational factors in shaping the pathologies of care, including how these influence the performance of patient-centered practices. Additionally, future research can examine the impact of leadership and organizational culture on the presence of the pathologies of care.

Finally, future research should replicate these findings in other ICU settings, to expand the understanding of each pathology, its prevalence, and then be able to evaluate strategies to mitigate pathologies of care. Understanding how these strategies can be systematically integrated into the ICU workflow could promote the goal of patient and person-centered care in the ICU.

## Conclusion

This study provides a clinician-informed account of how pressures inherent to ICU practice may give rise to interconnected patterns of care, described as hurry, blur, burden, and cruelty. By highlighting how these processes interact within everyday practice, the findings offer a lens to better understand and potentially address challenges to patient-centered care in intensive care settings.

## Data Availability

The datasets presented in this article are not readily available because the datasets consist of qualitative focus group transcripts that may contain potentially identifiable information. Due to privacy and confidentiality considerations, the data are not publicly available but may be shared in de-identified form upon reasonable request and with appropriate institutional approvals. Requests to access the datasets should be directed to MA, alzahidy.misk@mayo.edu.

## Supplementary material

Supplementary Material S1 Semi-structured focus group discussion guide.

## References

[1] Kunneman M Griffioen IPM Labrie NHM Kristiansen M Montori VM Van Beusekom MM. Making care fit manifesto. BMJ Evid Based Med. (2023) 28(1):5–6. 10.1136/bmjebm-2021-111871 34815303 PMC9887358

[2] Epstein RM Street RL Jr. The values and value of patient-centered care. Ann Fam Med. (2011) 9(2):100–3. 10.1370/afm.1239 21403134 PMC3056855

[3] Allwood D Koka S Armbruster R Montori V. Leadership for careful and kind care. BMJ Lead. (2022) 6(2):125–9. 10.1136/leader-2021-000451 36170535 PMC12038097

[4] Montori VM Allwood D. Careful, kind care is our compass out of the pandemic fog. Br Med J. (2022) 379:e073444. 10.1136/bmj-2022-073444 36517069

[5] Poplau S Linzer M Allwood D Montori V Armbruster R Koka S. Designing the careful and kind clinic: an evidence-based approach. BMJ Lead. (2022) 6(2):87–91. 10.1136/leader-2021-000538 36170536

[6] Gopalan PD Pershad S. Decision-making in ICU – a systematic review of factors considered important by ICU clinician decision makers with regard to ICU triage decisions. J Crit Care. (2019) 50:99–110. 10.1016/j.jcrc.2018.11.027 30502690

[7] Yee A. Clinical decision-making in the intensive care unit: a concept analysis. Intensive Crit Care Nurs. (2023) 77:103430. 10.1016/j.iccn.2023.103430

[8] Basile MJ Rubin E Wilson ME Polo J Jacome SN Brown SM. Humanizing the ICU patient: a qualitative exploration of behaviors experienced by patients, caregivers, and ICU staff. Crit Care Explor. (2021) 3(6):e0463. 10.1097/CCE.0000000000000463 34151284 PMC8208441

[9] Nielsen AH Kvande ME Angel S. Humanizing and dehumanizing intensive care: thematic synthesis (HumanIC). J Adv Nurs. (2023) 79(1):385–401. 10.1111/jan.15477 36281216 PMC10092106

[10] Law AC Roche S Reichheld A Folcarelli P Cocchi MN Howell MD. Failures in the respectful care of critically ill patients. Jt Comm J Qual Patient Saf. (2019) 45(4):276–84. 10.1016/j.jcjq.2018.05.008 30170754 PMC6657523

[11] Jakimowicz S Perry L Lewis J. Insights on compassion and patient-centred nursing in intensive care: a constructivist grounded theory. J Clin Nurs. (2018) 27(7-8):1599–611. 10.1111/jocn.14231 29266484

[12] Corrêa M Castanhel FD Grosseman S. Patients’ perception of medical communication and their needs during the stay in the intensive care unit. Rev Bras Ter Intensiva. (2021) 33(3):401–11. 10.5935/0103-507X.20210050 35107551 PMC8555396

[13] Lebet RM Hasbani NR Sisko MT Agus MSD Nadkarni VM Wypij D. Nurses’ perceptions of workload burden in pediatric critical care. Am J Crit Care. (2021) 30(1):27–35. 10.4037/ajcc2021725 33385203

[14] Lin KH Selvanayagam N Patnaik S Kuo CY. Burnout among physicians and nurses working in intensive care units and emergency departments: a systematic review and meta-analysis. J Emerg Nurs. (2025) 51(4):702–20. 10.1016/j.jen.2025.02.007 40088246

[15] Edú-Valsania S Laguía A Moriano JA. Burnout: a review of theory and measurement. Int J Environ Res Public Health. (2022) 19(3):1780. 10.3390/ijerph19031780 35162802 PMC8834764

[16] Reader TW Gillespie A. Patient neglect in healthcare institutions: a systematic review and conceptual model. BMC Health Serv Res. (2013) 13:156. 10.1186/1472-6963-13-156 23631468 PMC3660245

[17] O'Brien BC Harris IB Beckman TJ Reed DA Cook DA. Standards for reporting qualitative research: a synthesis of recommendations. Acad Med. (2014) 89(9):1245–51. 10.1097/ACM.0000000000000388 24979285

[18] Ahmad SR Rhudy L Barwise AK Ozkan MC Gajic O Karnatovskaia LV. Perspectives of clinicians on the value of the get to know me board in the ICU. Chest. (2025) 167(2):561–70. 10.1016/j.chest.2024.10.016 39427707

[19] Gajic O Anderson BD. “Get to know me” board. Crit Care Explor. (2019) 1(8):e0030. 10.1097/CCE.0000000000000030 32166271 PMC7063957

[20] McDonald KM Rodriguez HP Shortell SM. Organizational influences on time pressure stressors and potential patient consequences in primary care. Med Care. (2018) 56(10):822–30. 10.1097/MLR.0000000000000974 30130270 PMC6402989

[21] Rolfsen ML Ely EW. What's the “get to know me board”? Chest. (2025) 167(2):316–8. 10.1016/j.chest.2024.11.028 39939054

[22] Shah D Ghosh K Singh R Bonfante I Nagales J Wuthrich A. Picturing empathy in the intensive care unit: patient photographs at an urban community teaching hospital. Am J Crit Care. (2024) 33(6):455–61. 10.4037/ajcc2024637 39482089

[23] De La Fuente-Martos C Rojas-Amezcua M Gómez-Espejo MR Lara-Aguayo P Morán-Fernandez E Aguilar-Alonso E. Humanization in healthcare arises from the need for a holistic approach to illness. Med Intensiva (Engl Ed). (2018) 42(2):99–109. 10.1016/j.medin.2017.08.002 29132912

[24] Montori V Hargraves I Breslin M. Careful and kind care requires unhurried conversations. Catalyst Carryover. (2019) 5(5).

[25] Nin Vaeza N Martin Delgado MC Heras La Calle G. Humanizing intensive care: toward a human-centered care ICU model. Crit Care Med. (2020) 48(3):385–90. 10.1097/CCM.0000000000004191 32058373

[26] Reifarth E Garcia Borrega J Kochanek M. How to communicate with family members of the critically ill in the intensive care unit: a scoping review. Intensive Crit Care Nurs. (2023) 74:103328. 10.1016/j.iccn.2022.103328 36180318

[27] Epstein RM. Mindful practice. JAMA. (1999) 282(9):833–9. 10.1001/jama.282.9.833 10478689

[28] Lee C Britto S Diwan K. Evaluating the impact of artificial intelligence (AI) on clinical documentation efficiency and accuracy across clinical settings: a scoping review. Cureus. (2024) 16(11):e73994. 10.7759/cureus.73994 39703286 PMC11658896

[29] Guest G Namey E Taylor J Eley N McKenna K. Comparing focus groups and individual interviews: findings from a randomized study. Int J Soc Res Methodol. (2017) 20(6):693–708. 10.1080/13645579.2017.1281601

[30] Parsons M Greenwood J. A guide to the use of focus groups in health care research: part 1. Contemp Nurse. (2000) 9(2):169–80. 10.5172/conu.2000.9.2.169 11855006

