# Supplementary material for: Humanizing care in the ICU: how system pressures shape interacting patterns of hurry, blur, burden, and cruelty
Source: Front Health Serv. 2026 Aug 11;6:1867800. doi: 10.3389/frhs.2026.1867800 (PMC13503235; doi:10.3389/frhs.2026.1867800)
Supplement: Supplementary file 1 [file Table1.docx]

**Supplemental Material 1**

**Semi-structured discussion guide**

**Opening**

Welcome everyone, thank them for their attendance, introduce self and staff, confirm with everyone that the session may be recorded, but transcripts will be de-identified.

The purpose of the project overall is to understand problems in health care described by Dr. Montori and the KER unit, also referred to as ‘pathologies in healthcare’, if relevant in the ICU. What we are asking of you today is to share your thoughts and experiences regarding these pathologies. This project is funded by the Critical Care Research Subcommittee. Participation is voluntary and your participation or lack of will not affect current or future care or employment at Mayo Clinic. There is no compensation for participating and while we do not expect you to experience any risks or benefits from this work, your insight may help improve care for patients in the future.

**Ground Rules**

Set out ground rules:

1. What happens in the focus group stays in the focus group.
   a. Group members may share sensitive patient information, and we must agree to respect the confidentiality of this information.
2. One at a time.
   a. Please respect the thoughts, opinions, and stories of the other group members. When a member is talking, if you have something to say, write it down and wait until they are finished reflecting on what they have said. Be respectful, but conversational.
3. There are no wrong answers.
   a. We want to hear about your thoughts and experiences. These are very valuable to us and there is no wrong answer. We are not looking for consensus or only positive experiences or feedback. We want to explore a diversity of opinions. We want your honest opinion, and this will not affect your employment in any way.
4. Be respectful/No judgments.
   a. Sharing can be hard but remember there are no wrong answers. Please respect others’ stories and opinions and please only use their experiences to compare and contrast to your own and avoid making judgements about another’s thoughts, opinions, or story.
5. Any additional rules the group would like to add?

Confirm agreement to the ground rules and clarify that everyone understands and doesn’t have any questions regarding the ground rules.

**Questions**

During the brainstorming sessions we identified instances of problems in healthcare including hurry, blur, burden and cruelty. I have a few questions to help us better understand each of these problems.

**Opening Question**

1. As we begin, put in the chat:
   a. Name
   b. Practice area/location/role

**Introductory Question – Ideal State**

2. Before we discuss the pathologies, how might a patient describe the optimal ICU experience? That is, in an ideal world, what words or phrases might a patient or their family use to describe an ICU stay?

**Hurry**

3. Now, moving to ways a patient’s ICU stay can be less than optimal, let’s discuss hurry. What does this mean in the ICU setting?
   a. In our brainstorming sessions we heard clinicians describe the ICU as hurried and mentioned that they often felt a sense of urgency.
      i. What might contribute to this sense of urgency?
      ii. How might this urgency affect patient care or the patient's experience of care?
          1. Lack of personalization?
          2. Non-English speakers?
      iii. How might we reduce the sense of urgency clinicians feel in the ICU?

**Burden**

4. Moving on from hurry, let’s discuss burden. Burden could be to the patient, the family member, and the clinician. What does this mean in the ICU setting?
   a. Clinicians in our brainstorming sessions described being overwhelmed.
      i. What might contribute to this sense of being overwhelmed?
         1. Staff shortages?
         2. Administrative work?
         3. Stress?
      ii. How might clinicians’ feeling of being overwhelmed affect patient care or the experience of care?
      iii. How might we reduce clinicians’ feeling of being overwhelmed in the ICU?

**Blur**

5. Next, I want to talk about blur. One way of understanding blur is not seeing the patient in ‘high definition’. What does this mean in the ICU setting?
   a. Clinicians in our brainstorming sessions described occurrences of blur.
      i. What might contribute to blur in the ICU?
         1. Information overload?
      ii. How might blur affect patient care or the experience of care?
      iii. How might we reduce the occurrence of blur in the ICU?

**Cruelty**

6. Finally, let’s discuss cruelty. What does this mean in the ICU setting?
   a. Clinicians in our brainstorming sessions described situations where care lacked compassion or sensitivity.
      i. What might contribute to cruelty in the ICU?
      ii. How might cruelty affect patient care or the experience of care?
          1. Early morning blood draws
      iii. How might we reduce the occurrence of cruelty in the ICU?

**Ending Question**

7. As we begin to wrap up, I want to ask a few closing questions.
   a. Of the pathologies we talked about today (hurry, burden, blur, and cruelty), which do you think has the most impact on either patient care or the patient experience?
   b. What is the most important thing that can be done to address the pathologies of care in the ICU?

**Summary Question**

8. Today we discussed how the pathologies of care manifest in the ICU. We heard that <describe briefly the main findings that you heard participants say>. How well does that capture what was said?
   a. Anything missing?

**Closing**

Thank you all for your participation. Please remember the ground rules. The next steps are that we will interview patients and explore how these pathologies look like from their perspective.
